# Mismatch repair deficiency/microsatellite instability-high as a predictor for anti-PD-1/PD-L1 immunotherapy efficacy

**DOI:** 10.1186/s13045-019-0738-1

**Published:** 2019-05-31

**Authors:** Pengfei Zhao, Li Li, Xiaoyue Jiang, Qin Li

**Affiliations:** 1 Department of Radiotherapy, Beijing Friendship Hospital, Capital Medical University, Beijing, 100050 China; 2 Department of Oncology, Beijing Friendship Hospital, Capital Medical University, Beijing, 100050 China

**Keywords:** Tumor, Mismatch repair deficiency, Microsatellite instability, Immunotherapy, Immune checkpoint blockade

## Abstract

Immunotherapies have led to substantial changes in cancer treatment and have been a persistently popular topic in cancer research because they tremendously improve the efficacy of treatment and survival of individuals with various cancer types. However, only a small proportion of patients are sensitive to immunotherapy, and specific biomarkers are urgently needed to separate responders from nonresponders. Mismatch repair pathways play a vital role in identifying and repairing mismatched bases during DNA replication and genetic recombination in normal and cancer cells. Defects in DNA mismatch repair proteins and subsequent microsatellite instability-high lead to the accumulation of mutation loads in cancer-related genes and the generation of neoantigens, which stimulate the anti-tumor immune response of the host. Mismatch repair deficiency/microsatellite instability-high represents a good prognosis in early colorectal cancer settings without adjuvant treatment and a poor prognosis in patients with metastasis. Several clinical trials have demonstrated that mismatch repair deficiency or microsatellite instability-high is significantly associated with long-term immunotherapy-related responses and better prognosis in colorectal and noncolorectal malignancies treated with immune checkpoint inhibitors. To date, the anti-programmed cell death-1 inhibitor pembrolizumab has been approved for mismatch repair deficiency/microsatellite instability-high refractory or metastatic solid tumors, and nivolumab has been approved for colorectal cancer patients with mismatch repair deficiency/microsatellite instability-high. This is the first time in the history of cancer therapy that the same biomarker has been used to guide immune therapy regardless of tumor type. This review summarizes the features of mismatch repair deficiency/microsatellite instability-high, its relationship with programmed death-ligand 1/programmed cell death-1, and the recent advances in predicting immunotherapy efficacy.

**Electronic supplementary material:**

The online version of this article (10.1186/s13045-019-0738-1) contains supplementary material, which is available to authorized users.

In recent years, immunotherapy has become the focus of the revamped cancer therapeutic paradigm. Immunotherapy has brought amazing and long-lasting tumor remission for several common solid malignancies and refractory malignancies [1–6]. Nonetheless, the extensive clinical application of immunotherapies has been limited because some tumors show relatively poor efficacy and low response rates [1, 2]. Therefore, biomarkers are urgently needed to distinguish the sensitive patients and to predict therapeutic response. Ample evidence supports programmed death-ligand 1 (PD-L1) or programmed cell death-1 (PD-1) expression, tumor mutational burden (TMB), numbers of tumor-infiltrating lymphocytes (TILs), peripheral blood lymphocyte count, mismatch repair deficiency (dMMR), and microsatellite instability-high (MSI-H) as predictive biomarkers that guide the clinical application of immune checkpoint blockade (ICB) therapies [7]. Among many indicators, dMMR and MSI-H show unique advantages. Tumors with dMMR or MSI-H are sensitive to ICB, particularly to PD-1 and PD-L1 inhibitors. It is worth emphasizing that dMMR or MSI-H could identify responders regardless of tumor location and tumor type, that is, they have the ability to guide different tumor immunotherapies in the same manner. Subsequently, the US Food and Drug Administration (FDA) approved the indication of ICB for all dMMR/MSI-H solid tumors [8]. In this review, we elaborate on the expression of MMR/MSI in multiple tumors, the predictive value of MMR/MSI-H in ICB treatment, the relationship between MSI-H and other predictor markers.

## Mismatch repair proficiency/deficiency and microsatellite instability

**Fig. 1:**
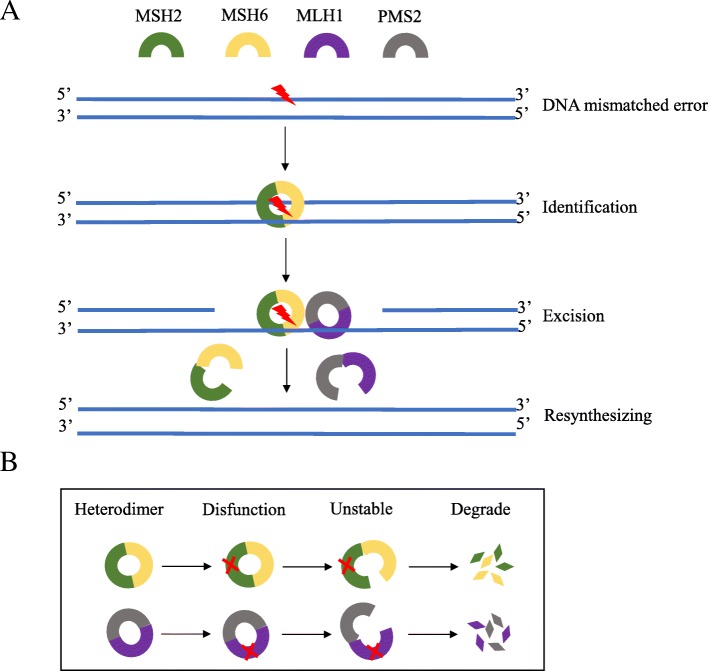
The process of DNA mismatch repair

The DNA mismatch repair (MMR) system, which exists extensively in organisms from prokaryotes to eukaryotes, is a highly conserved repair mechanism in cellular evolution. MMR was first found as a causative germline alteration in patients with Lynch syndrome in 1993 and was termed a microsatellite [9–12]. The MMR system plays key roles in identifying and repairing mismatched nucleotides during genetic recombination or as a result of damage caused by external physical or chemical insults. MMR guarantees genomic integrity and stability and avoids insertions and deletions of abnormal DNA at microsatellites. The MMR system comprises a series of specific DNA mismatch repair enzymes and is usually dependent on four key genes: mutL homologue 1 (*MLH1*), postmeiotic segregation increased 2 (*PMS2*), mutS homologue 2 (*MSH2*), and mutS 6 (*MSH6*). MLH1, PMS2, MSH2, and MSH6 proteins are mainly detected through immunohistochemical methods in the clinic. MSH2/MSH6 heterodimers are responsible for binding to the initial DNA mismatched base errors (including single-base mismatch and incorrect insertion or deletion loop mismatch) by conformational changes, and MLH1/PMS2 heterodimers are in charge of the excision and synthesis of corrected DNA chains in the mismatch site (see Fig. 1a). If one or more proteins are not expressed or are dysfunctional, the status is called dMMR; otherwise, the status is considered mismatch repair proficient (pMMR). MLH1 and MSH2 play pivotal roles in the process of MMR by dimerizing and interacting with MSH6 and PMS2. The dysfunction of MLH1 or MSH2 leads to the inactivation of MLH1/PMS2 or MSH2/MSH6 and the degradation of PMS2 or MSH6 (see Fig. 1b). Lynch syndrome is a common hereditary disease that is characterized by germline mutations in *MMR* genes [13]. Lynch syndrome is associated with multiple cancers, especially colon cancer and endometrial cancer. A lack of *MSH2*, substantial mutations in the *MLH1* or *MSH2* genes, *MLH1*-methylation inactivation, and transcriptional silencing lead to Lynch syndrome [14, 15]. Deletion mutations in *MLH1* and *MSH2* account for 42–50% and 33–39%; however, *MSH6* and *PMS2* mutations account for only 7–18% and less than 7%, respectively [16–19]. A hypothesis that heterozygous germline deletions in the epithelial cell adhesion molecule (*EPCAM*) gene as one factor that leads to *MSH2* defects has been confirmed, and the addition of *EPCAM* to the diagnostic panel for Lynch syndrome in *MSH2*-defective tumors has been advised [19, 20].

The inactivation of *MMR* genes and MMR protein dysfunction may be the results of germline mutations or spontaneous hypermutation alterations, which may induce microsatellite instability (MSI). More than 100,000 areas of short tandem repetitive DNA sequences are the diagnostic sites of MSI. Two mononucleotide repeats (*BAT25* and *BAT26*) and three dinucleotide repeats (*D5S346*, *D2S123*, and *D17S250*) are the standard sites in panels for MSI testing, as recommended by the National Cancer Institute in 1998 [21–23]. If two or more repeats are altered, the tumor is defined as MSI-H; if only one mutated sequence is found, the tumor is considered to be microsatellite instability-low (MSI-L). Otherwise, it is said to have microsatellite stability (MSS). There is a high level of consistency (almost 90–95%) between dMMR and MSI-H among many tumors; therefore, these two terms are used almost interchangeably [24]. MSI-H or dMMR has been widely detected and reported in Lynch syndrome-associated tumors, usually in colorectal cancer (CRC) [12], gastrointestinal adenocarcinoma [23], and endometrial cancer [25]. However, MSI-H or dMMR rarely appears in breast cancer [26], prostate cancer [27], and lung adenocarcinoma [9]. MSI-H varies from 0–31.37% in 39 cancer types. Endometrial carcinoma of the uterine corpus, colon adenocarcinoma, and stomach adenocarcinoma rank in the top 3 in terms of the prevalence of MSI-H, followed by rectal adenocarcinoma, adrenocortical carcinoma, and uterine carcinosarcoma. MSI-H has not been detected in more than ten tumors, as shown in Table 1 [28–30]. The prevalence of MSI-H in these studies was mostly derived from tissues of early-stage tumors [28–30]. Le DT et al. reported that dMMR occurred more often in early-stage tumors than in late-stage tumors (stage IV) [30]. Venderbosch [31] also found that the incidence of dMMR in metastatic CRC was 5%, which was lower than that (19.72%) in early-stage CRC. MOSAIC, MANTIS, and next-generation sequencing were used to analyze MSI status [27–29], as sensitive standardized detection of MSI is necessary.

**Table 1:** Prevalence of MSI-H in 39 cancer types

Cancer type	MSI-H (%)	Cancer type	MSI-H (%)	Cancer type	MSI-H (%)	Cancer type	MSI-H (%)
UCEC	17.00–31.37	BRCA	0.00–1.53	PRAD	0.60–3.00	KICH	0.00
COAD	6.00–19.72	KIRC	1.47	LUAD	0.53–1.00	KIRP	0.00
STAD	9.00–19.09	OV	1.37–2.00	BLCA	0.49	LAML	0.00
READ	5.73	CHOL	1.35–3.00	NBL	0.45	NPC	0.00
ACC	4.35	THYM	0.81	LGG	0.39	PAAD	0–2.00
UCS	3.00–3.51	LIHC	0.80–3.00	CLL	0.30	PCPG	0.00
CESC	2.62–4.00	HNSC	0.78	GBM	0.25	TGCT	0.00
WT	2.44	SARC	0.78	AML	0.00	THCA	0.00–3.00
MESO	2.41	SKCM	0.00–0.64	CTCL	0.00	UVM	0.00–2.00
ESCA	1.63	LUSC	0.60	DLBC	0.00		

*Abbreviations*: *UCEC* uterine corpus endometrial carcinoma, *COAD* colon adenocarcinoma, *STAD* stomach adenocarcinoma, *READ* rectal adenocarcinoma, *ACC* adrenocortical carcinoma, *UCS* uterine carcinosarcoma, *CESC* cervical squamous cell carcinoma and endocervical adenocarcinoma, *WT* Wilms tumor, *MESO* mesothelioma, *ESCA* esophageal carcinoma, *BRCA* breast carcinoma, *KIRC* kidney renal clear cell carcinoma, *OV* ovarian serous cystadenocarcinoma, *CHOL* cholangiocarcinoma, *THYM* thymoma, *LIHC* liver hepatocellular carcinoma, *HNSC* head and neck squamous cell carcinoma, *SARC* sarcoma, *SKCM* skin cutaneous melanoma, *LUSC* lung squamous cell carcinoma, *PRAD* prostate adenocarcinoma, *LUAD* lung adenocarcinoma, *BLCA* bladder carcinoma, *NBL* pediatric neuroblastoma, *LGG* lower-grade glioma, *CLL* chronic lymphocytic leukemia, *GBM* glioblastoma multiforme, *AML* pediatric acute myeloid leukemia, *CTCL* cutaneous T cell lymphoma, *DLBC* diffuse large B cell lymphoma, *KICH* kidney chromophobe, *KIRP* kidney renal papillary cell carcinoma, *LAML* acute myeloid leukemia, *NPC* nasopharyngeal carcinoma, *PAAD* pancreatic adenocarcinoma, *PCPG* pheochromocytoma and paraganglioma, *TGCT* testicular germ cell tumor, *THCA* thyroid carcinoma, *UVM* uveal melanoma

## The relationship between MMR and multiple tumors

**Fig. 2:**
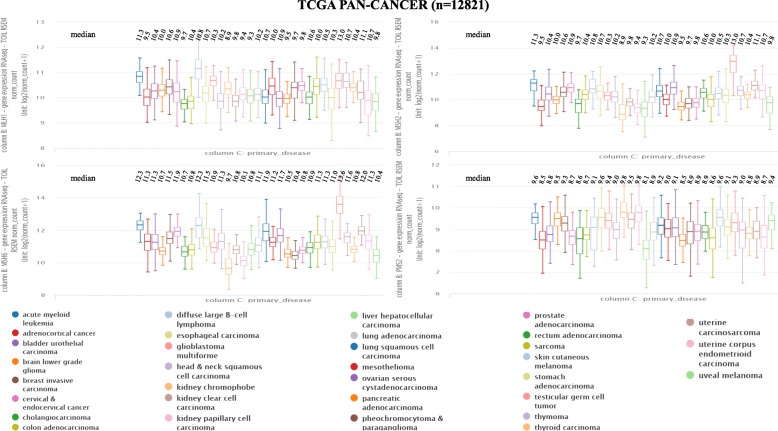
Expression of mismatch repair proteins in 33 tumors

**Fig. 3:**
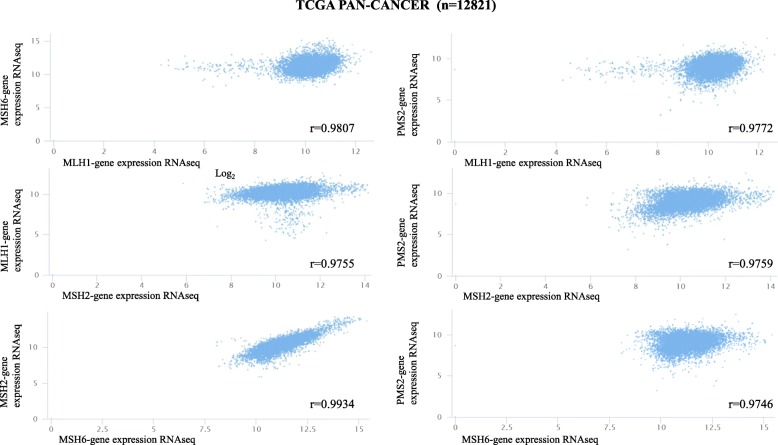
Correlation of *MLH1*, *PMS2*, *MSH2*, and *MSH6* expression in 12,821 tumor samples

In this review, *MMR* gene expression of 12,821 samples from 33 different tumors were pooled and analyzed through The Cancer Genome Atlas (TCGA) database (http://www.cbioportal.org/). Data from all TCGA cohorts were combined to produce this PanCancer dataset. Values of gene expression from the RNAseq experiment shown in Fig. 2 are log_2_(*x* + 1) transformed RSEM values. The expression of *MMR* genes is different in many tumors and even in the same tumor. Generally, the expression of *MSH6* is almost always the highest, while *PMS2* expression is the lowest. *MLH1* gene expression is observed more often in acute myeloid leukemia, glioblastoma multiforme, and testicular germ cell tumors. *MSH6* and *MSH2* are expressed more frequently in acute myeloid leukemia, testicular germ cell tumors and uterine carcinosarcoma. However, *PMS2* gene expression in the kidney chromophobe and kidney papillary cell carcinoma is higher than in other tumors (see Fig. 2). The correlations among *MLH1*, *PMS2*, *MSH2*, and *MSH6* were also demonstrated using the TCGA. Excellent positive correlations were observed among the four *MMR* genes (all *r* > 0.97) (see Fig. 3). The good correlations coincide with the heterodimeric characteristic of these four genes.

The survival analysis based on the TCGA suggests a significant association between the expression of *MMR* genes and prognosis in several tumors. In general, dMMR is correlated with an improved median overall survival (mOS) in most tumors other than head and neck cancer and pancreatic cancer. *MLH1−* is a prognostic factor in esophageal carcinoma and liver hepatocellular carcinoma (*P* = 0.049, 0.039, respectively), and *MSH2−* is correlated with improved mOS of bladder urothelial carcinoma, liver hepatocellular carcinoma, prostate adenocarcinoma, and sarcoma (*P* = 0.029, 0.002, 0.011, and 0.001, respectively). The mOS of the *MSH6−* group is prolonged compared with that of the *MSH6+* group in patients with bladder urothelial carcinoma (*P* = 0.015). Except for pancreatic cancer, *PMS2−* patients exhibit no survival benefit in most cancers (see Additional file 1: Figure S1). A comprehensive analysis indicated that mOS was improved only in esophageal carcinoma and liver hepatocellular carcinoma patients with four *MMR* gene deficiencies (*P* = 0.049 and 0.001, respectively) (see Additional file 2: Figure S2).

## The predictive value of dMMR/MSI-H in multiple tumors

A strong clinical relationship is observed between MMR status and CRC. dMMR/MSI-H occurs in up to 15% of sporadic colon cancers [32]. Several clinicopathological variables, such as proximal tumor location, advanced age (> 65 years), poor differentiation, diploid DNA content, and the *BRAF* V600E mutation were found to be associated with the prevalence of MSI-H [32]. *MLH1* dysfunction is responsible for most tumors in sporadic CRC patients with dMMR because its transcriptional promoter gene is silenced due to CpG island hypermethylation. The prognostic and predictive values of MMR in CRC are different in early-stage and late-stage. Most results indicated that dMMR is a positive prognostic factor in early-stage (II/III) rather than late-stage (IV) [30, 31, 33–36]. Ribic et al. [33] observed that patients with MSI-H had significant increased a 5-year survival rate compared with the MSI-L or MSS counterparts with stage II or stage III CRC who did not receive fluorouracil-based adjuvant chemotherapy (88.0% versus 66.4%, *P* = 0.004), but the 5-year survival rate of the former group was not significantly increased in patients who received adjuvant chemotherapy (70.7% versus 75.5%, *P* = 0.66). Guastadisegni [34] demonstrated that the odds ratio (OR) for OS associated with MSI-H was 0.6 (*P* < 0.0001) in patients with stages I–IV and 0.65 (*P* < 0.0001) in patients with stages II–III CRC and that the OR for disease-free survival (DFS) was 0.58 (*P* < 0.0001). They pooled the data and showed that MSS patients treated with 5-fluorouracil had a better prognosis but that the benefit was not obvious for MSI-H CRC patients (OR 0.52, 95% CI 0.4–0.6, *P* < 0.0001 versus OR 0.69, 95% CI 0.3–1.5, *P* = 0.10). Sargent et al. [35] also concluded that patients with stages II–III CRC with pMMR exhibited improved DFS (hazard ratio [HR] 0.67, 95% CI 0.48–0.93, *P* = 0.02) resulting from adjuvant therapy compared with those who underwent surgery alone. Regarding the predictive value of MMR status to the response to adjuvant irinotecan- or oxaliplatin-based treatment in stage III CRC patients is contradictory [37–40]. MSI-H or dMMR has a good prognostic predictive value in early-stage CRC settings without adjuvant chemotherapy and plays a negative predictive role for adjuvant fluorouracil-based chemotherapy in patients with resected CRC. However, the value of dMMR/MSI-H in metastatic CRC (mCRC) is opposite to that of early-stage CRC. A meta-analysis confirmed that mCRC patients with dMMR had poorer survival compared with pMMR patients, which might be due to a *BRAF* V600E mutation. The median progressive-free survival (mPFS) was 6.2 months in dMMR patients and 7.6 months in pMMR patients (HR 1.33, *P* = 0.001), while the mOS was 13.6 months and 16.8 months, respectively (HR 1.35, *P* = 0.001) [31]. The study of Mayo Clinic showed that mCRC patients with MSI-H had earlier disease recurrence (12.9 months vs. 20.9 months, *P* = 0.034) and poorer OS (28.1 months vs. 37.4 months, *P* = 0.99) than MSS patients [36]. Detection of a *BRAF* V600E mutation is recommended in sporadic MSI tumors with silenced *MLH1*, as the presence of a *BRAF* V600E mutation strongly suggests that the etiology of the disease is sporadic [41].

The predictive value of dMMR was also investigated in other tumors. dMMR has been demonstrated in 20–40% of endometrial cancers [25, 42], but data on its prognostic value are controversial [25, 43, 44]. A meta-analysis including 23 studies found no association between OS (HR 2.0, *P* = 0.11) or DFS (HR 1.31, *P* = 0.66) and dMMR [45] in endometrial cancer. MSI frequency or dMMR expression in ovarian tumors varied from 5–13% [46–48] in MSI patients and from 2–29% in dMMR patients [49]. dMMR as a positive predictive biomarker for survival or response in ovarian cancers has not been confirmed [50, 51]. In gastric cancer (GC), MSI-H has been identified in approximately 10–20% of tumors, and patients with MSI-H demonstrate an improved survival compared with patients with MSS [52, 53]. However, data on the predictive value of MSI for GC patients treated with fluorouracil-based chemotherapy are conflicting [54, 55]. In a study on melanoma, the expression of MSI was increased when the disease progressed from benign to malignant and then to metastatic [56]. Related studies in other tumors are few in number and limited, and therefore, evidence of the prognostic value is insufficient.

## dMMR/MSI-H predicts the efficacy of anti-PD-1/PD-L1 immunotherapy

Anti-PD-1/PD-L1 immunotherapies have led to tremendous success in treating certain cancers, such as melanoma, non-small cell lung cancer (NSCLC), mCRC, renal cell carcinoma, bladder cancer, head and neck squamous cell carcinoma, classical Hodgkin’s lymphoma, and Merkel cell carcinoma [1–6, 57–63]. However, only a small fraction of patients with these malignancies are sensitive to ICB therapies. For patients with NSCLC or metastatic melanoma [1, 2, 60], only 15.2–20% of patients who received single ICB therapy achieved objective response and 33.2–49% of patients obtained disease control. The precise recognition of targeted patients has always been the common goal of the researchers. Recent works suggest that MMR status could serve as a candidate biomarker and predict the responses of patients with solid tumors to ICB, irrespective of cancer type. Impressive results of pembrolizumab in patients with dMMR or MSI-H tumors after progression from prior chemotherapies have been shown in the KEYNOTE-016, 164, 012, 028, and 158 trials [8]. 149 patients with 15 different MSI-H or dMMR tumors were enrolled in the five trials. Patients with MSI-H/dMMR non-CRC were prospectively enrolled in single-arm trials (KEYNOTE-016, 158) or retrospectively identified in multi-cohort trials (KEYNOTE-012, 028) or in patients with one of 10 rare tumor types (KEYNOTE-158). 28 patients with CRC and 30 patients with non-CRC were included in KEYNOTE-016 trial, and 61 patients with CRC were enrolled in the initial interim analysis of KEYNOTE-164 trials. There were 6, 5, and 19 patients enrolled in KEYNOTE-012, 028, and 158 trials, respectively. Patients received pembrolizumab at 200 mg intravenously every 3 weeks in KEYNOTE-164, 158, and at 10 mg/kg intravenously every 2 weeks in KEYNOTE-028, 016, and 012 for 2 years until rapid disease progression or severe toxicity. The summarized results showed the overall response rate (ORR) was 39.6% (95% CI 31.7–47.9); moreover, the duration of response ranged from 1.6 to 27 months, with 78% of responses sustaining longer than 6 months [8, 64]. Pembrolizumab led to a durable response and survival benefits in patients with dMMR chemotherapy-refractory progressive metastatic tumors. Based on the above results, in 2017, the FDA accelerated the approval of pembrolizumab as a second- or higher-line choice for the treatment of patients with unresectable or metastatic dMMR/MSI-H solid tumors, irrespective of tumor type or site. This approval for a drug indication is noteworthy because it is the first time that one biomarker defined an indication regardless of the primary tumor site. However, further clinical trials consisting of sufficient number of patients and adequate follow-up are necessary to verify the efficacy of pembrolizumab in patients with dMMR/MSI-H.

As the partial published results of the KEYNOTE-016 trial, 41 patients were enrolled and assigned to the CRC with dMMR (11 patients), CRC with pMMR (21 patients), and non-CRC with dMMR (9 patients) groups. The outcome showed that the immune-related ORR and PFS rates were 40% and 78% in dMMR CRC patients, 71% and 67% in dMMR non-CRC patients, and 0% and 11% in pMMR cancers. Both the mPFS and mOS were not reached in dMMR CRC patients in contrast to the 2.2 and 5.0 months observed in pMMR CRC patients [65]. The KEYNOTE-164 and 158 trials both reported the positive evidence to support the FDA approval of pembrolizumab. The relative update data are showed in the following description. Sixty-three patients with unresectable locally advanced or metastatic dMMR/MSI-H CRC after the failure of prior fluoropyrimidine-, oxaliplatin-, and irinotecan-based chemotherapy were enrolled in the KEYNOTE-164 trial, and 77 patients with any advanced MSI-H solid tumors after one or more prior regimen, except CRC, were enrolled in the KEYNOTE-158 trial. Both trials indicated similar objective response rates (ORRs) (32% and 37.7%), mPFS (4.1 months and 4.3 months), and 6-month OS rates (87% and 73%) [66, 67]. In the KEYNOTE-164 trial, the 12-month PFS rate was 41% and the 12-month OS rate was 76%.

The efficacy of nivolumab was also investigated in patients with dMMR/MSI-H tumors. The open-label, multicenter, phase II study CheckMate 142 evaluated the efficacy of nivolumab in 74 mCRC patients with dMMR/MSI-H tumors that progressed during or after one-line conventional chemotherapy. Patients received 3 mg/kg nivolumab intravenously every 2 weeks until progressive disease, unacceptable toxicity, or others. In all, 31.1% of patients achieved an objective response, 69% of patients had disease control for more than 12 weeks, and the PFS and OS rates at 12 months were 50% and 73%, respectively. The medium duration of responses was not reached until a median follow-up of 12 months [68]. Based on this meaningful and durable clinical benefit, nivolumab was approved by the FDA as a new treatment option for mCRC patients with MSI-H or dMMR who had disease progression after chemotherapy with fluoropyrimidine, oxaliplatin, and irinotecan.

dMMR/MSI-H also predict the efficacy of ICB combined therapy. Studies have confirmed that the efficacy of nivolumab combined with ipilimumab is better than that of nivolumab alone in small cell lung cancer (SCLC) and melanoma [69, 70]. In the nivolumab plus ipilimumab cohort of the CheckMate 142 trial, 119 mCRC patients with dMMR/MSI-H who progressed after two or more prior therapies were enrolled. The results showed that the ORR was 55% and that the DCR for more than 12 weeks was 80%; the 12-month PFS rate was 71%, and the corresponding OS rate was 85% regardless of PD-L1 expression, *BRAF/KRAS* mutation status, or clinical history of Lynch syndrome (see Table 2) [71]. A preliminary result from H-J J Lenz et al. demonstrated that nivolumab plus low-dose ipilimumab had an inspiring and promising benefit as a first-line therapy for mCRC patients with dMMR/MSI-H. The ORR and DCR were 60% (27/45) and 84% (38/45), respectively. The PFS rate was 78%, and the OS rate was 83% at 12 months [72]. M Chalabi et al. conducted an exploratory phase II trial to investigate nivolumab plus ipilimumab as a neoadjuvant therapy in resectable, early-stage colon cancers with dMMR and pMMR. Seven dMMR and 8 pMMR patients were enrolled. A major pathological response was observed in 100% of the dMMR patients, but no response was observed in pMMR patients [73]. However, these trials were somewhat limited by the lack of random grouping and small sample size, and thus, further investigation is urgent and necessary (see Table 3).

**Table 2:** The relationship between ICB and dMMR/MSI-H

Author/year	Cancer type	*N*	Protocol	Results	PFS	OS
*Le DT* [65] 2015	dMMR CRC	41	Pembrolizumab 10 mg/kg, q14d, 20 weeks	ORR 40%	20-week PFS, 78%; mPFS: NR	mOS: NR
	pMMR CRC			ORR 0%	20-week PFS, 11%; mPFS: 2.2 months	mOS, 5.0 months
	dMMR non-CRC			ORR 71%	20-week PFS, 67%; mPFS: 5.4 months	mOS: NR
	pMMR CRC	25		ORR 0% DCR16%	mPFS, 2.4 months	mOS, 6 months
*Le DT* [30] 2017	12 tumors with dMMR	86	Pembrolizumab 10 mg/kg, q14d, 2 years	ORR 53% DCR 66%	12-month PFS, 64% 24-month PFS, 53%;mPFS: NR	12-month OS, 76% 24-month OS, 64%;mOS: NR
*Diaz LA* [66] 2017	Multiple types of solid tumors	77	Pembrolizumab 200 mg, q3w, 35 cycles	ORR 38% DCR 58%	6-month PFS, 45% mPFS, 4.3 months	6-month OS, 73%;mOS: NR
*D Le* [67] 2018	MMR/dMMR CRC	63	Pembrolizumab 200 mg, q3w, 35 cycles	ORR 28% DCR 51%	6-month PFS, 43% 12-month PFS, 41%;mPFS, 4.1 months	6-month OS, 87% 12-month OS, 76%;mOS: NR
*Overman MJ* [68] 2017	dMMR/MSI-H mCRC	74	Nivolumab 3 mg/kg, q2w, until PD	ORR 31% DCR 69% (≥ 12 weeks)	12-month PFS, 50% mPFS, 14.3 months	12-month OS, 73%;mOS: NR
*Overman MJ* [71] 2018	dMMR/MSI-H mCRC	119	Nivolumab 3 mg/kg + ipilimumab 1 mg/kg, q3w, 4 doses, followed by nivolumab 3 mg/kg, q2w, until PD	ORR 55% DCR 80%	9-month PFS, 76% 12-month PFS, 71%;mPFS: NR	9-month OS, 87% 12-month OS, 85%;mOS: NR
*H-J J Lenz* [72] 2018	dMMR/MSI-H mCRC	45	Nivolumab 3 mg/kg, q2w + ipilimumab 1 mg/kg, q6w, until PD (as first-line treatment)	ORR 60% DCR 84%	12-month PFS, 78%;mPFS: NR	12-month OS: 83%;mOS: NR
*M Chalabi* [73] 2018	dMMR early-stage CC pMMR early-stage CC	7 8	Nivolumab 3 mg/kg, d1, d15 + ipilimumab 1 mg/kg, d1	mPR 100% mPR 0%	NA	NA

*Abbreviations: dMMR* mismatch repair deficient, *CRC* colorectal cancer, *mCRC* metastatic colorectal cancer, *CC* colon cancers, *pMMR* mismatch repair proficient, *ORR* objective response rate, *PFS* progression-free survival, *mPFS* median progression-free survival, *NR* not reached (the responses were durable and the results were not reached until the end of follow-up), *mOS* median overall survival, *DCR* disease control rate, *OS* overall survival, *mCRC* metastatic colorectal cancer, *MSI-H* microsatellite instability-high, *PD* disease progression, *mPR* major pathological response, *NA* not available

**Table 3:** Ongoing clinical trials evaluating ICB therapies in dMMR/MSI-H tumors

Clinical trial	Phase	Drug treatment	Drugs	Tumor type	Current status
NCT03150706	II	Avelumab	Anti-PD-L1 mAb	Previously treated dMMR/MSI-H or POLE-mutated mCRC	Ongoing
NCT03435107	II	Durvalumab	Anti-PD-L1 mAb	Previously treated dMMR/MSI-H or POLE-mutated mCRC	Ongoing
NCT02983578	II	Durvalumab (MEDI4736) + AZD9150	Anti-PD-L1 mAb + Antisense STAT3	dMMR CRC, NSCLC, and advanced pancreatic cancer	Ongoing
NCT02997228	III	Atezolizumab + mFOLFOX6 + bevacizumab versus mFOLFOX6 + bevacizumab versus atezolizumab	Anti-PD-L1 mAb	dMMR mCRC	Ongoing
NCT02912559	III	Atezolizumab + standard chemotherapy* versus standard chemotherapy*	Anti-PD-L1 mAb	dMMR stage III resected CRC	Ongoing
NCT03257163	II	Pembrolizumab + capecitabine + radiation therapy	Anti-PD-1 mAb	dMMR and Epstein-Barr virus positive GC	Ongoing
NCT02563002	III	Pembrolizumab versus standard therapy**	Anti-PD-1 mAb	dMMR/MSI-H stage IV CRC	Not recruiting
NCT03236935	Ib	Pembrolizumab + L-NMMA	Anti-PD-1 mAb + nitric oxide synthase inhibitor	dMMR/MSI-H cancer, melanoma, NSCLC, HNSCC, classic HL, and urothelial carcinoma	Ongoing
NCT03607890	II	Nivolumab + relatlimab	Anti-PD-1 mAb + anti-LAG-3 mAb	MSI-H solid tumors refractory to prior PD-(L)1 therapy	Not recruiting
NCT02992964	I/II	Nivolumab	Anti-PD-1 mAb	Pediatric patients with hypermutant cancers, including biallelic MMR syndrome	Ongoing
NCT03241745	II	Nivolumab	Anti-PD-1 mAb	dMMR/MSI-H/hypermutated uterine cancer	Ongoing
NCT02060188	II	Nivolumab versus nivolumab + ipilimumab or nivolumab + ipilimumab + cobimetinib or nivolumab + BMS-986016 or nivolumab + daratumumab	Anti-PD-1 mAb + Anti-CTLA-4 mAb + MEK inhibitor + anti-LAG-3 mAb + anti-CD38 mAb	dMMR/pMMR/MSI-H/MSI-L/MSS CRC	Ongoing

*Abbreviations: ICBs* immune checkpoint blockades, *PD-L1* programmed death-ligand 1, *mAb* monoclonal antibody, *dMMR* mismatch repair deficient, *pMMR* mismatch repair proficient, *MSI-H* microsatellite instability-high, *MSI-L* microsatellite instability-low, *MSS* microsatellite stable, *CTLA-4* cytotoxic T-lymphocyte protein 4, *mCRC* metastatic colorectal cancer, *CRC* colorectal cancer, *NSCLC* non-small cell lung cancer, *GC* gastric cancer, *HNSCC* head and neck squamous cell carcinoma, *HL* Hodgkin lymphoma, *mFOLFOX 6* denotes fluorouracil plus leucovorin calcium and oxaliplatin

*Standard chemotherapy denotes fluorouracil plus leucovorin calcium and oxaliplatin

**Standard therapy denotes mFOLFOX6 or mFOLFOX6 plus bevacizumab, or mFOLFOX6 plus cetuximab, or FOLFIRI, or FOLFIRI plus bevacizumab, or FOLFIRI plus cetuximab (FOLFIRI denotes irinotecan plus leucovorin and fluorouracil)

Why does dMMR reflect the efficacy of immunotherapy? Le DT et al. reported that an average of 1782 somatic mutations per tumor and 578 potential neoantigens were found in dMMR tumors, compared with 73 mutations and 21 neoantigens in pMMR tumors by exome sequencing (*P* = 0.007). Higher numbers of somatic mutations and neoantigens were correlated with better responses and longer PFS. Furthermore, dMMR tumors have a dense infiltration of CD8^+^ TILs, which induce a better and more durable response [65]. In view of the abovementioned results, routine testing of the MMR status should be considered in the clinic regardless of tumor origin, which might bring an unexpected benefit to the patients with dMMR/MSI-H tumors. In summary, dMMR/MSI-H tumors treated with ICB demonstrate a durable response and a sustained survival benefit, and the combination of ICB therapies could further improve outcomes in these patients. The survival benefit remains to be explored in patients with refractory metastatic cancers. Table 3 shows the ongoing clinical trials evaluating ICB therapies in dMMR/MSI-H tumors. dMMR tumors are more frequent in early-stage cancers than in metastatic cancers, and therefore, the efficacy of immunotherapy in early-stage tumors is worthy of further investigation.

## Relationship between dMMR/MSI-H and other immune biomarkers

PD-1/PD-L1 checkpoints have important functions in maintaining immune-tolerance and preventing effective antitumor immunity. The numbers of PD-1^+^ TILs are significantly different among various cancer types and range from 0% in extraskeletal myxoid chondrosarcomas and uterine sarcoma to 93% in ovarian cancer [49, 74]. The positive rates of membranous PD-L1 expression vary from 50–97% in NSCLC, bladder carcinoma, renal cell carcinoma, endometrial cancer, melanoma, and sarcomas, but its expression is less than 10% in liver cancer and is absent in Merkel cell carcinoma [74]. PD-L1^+^ expression has been used as common stratification factor in many clinical trials and used as an important biomarker to guide the ICB therapy in clinical practice [61, 75–80]. PD-L1^+^ expression is closely related to dMMR/MSI-H status. Both Gatalica and Inaguma reported that the proportion of PD-L1^+^ expression in dMMR/MSI-H CRC was significantly higher than in pMMR/MSS CRC [74, 81]. Kim ST et al. reported that PD-L1^+^ expression was 38.9% in MLH1/MSH2-negative solid tumors compared with 15.2% in pMMR tumors [82]. In other studies, the PD-L1^+^ rate varied from 12.1–35.2% in pMMR GC and from 46.7–60.0% in dMMR GC (*P* < 0.01) [83, 84] (see Table 4). Lee SJ et al. [85] showed that the expression of PD-L1, lymphocyte-activation gene 3 (LAG3), and indolamine 2′3′-dioxygenase 1 (IDO1) in TILs was 68.6%, 13.5%, and 28.1%, respectively, in 89 patients with MSI-H colon cancer. A higher number of mutations in DNA coding sequences in MSI-H tumors have more potential to stimulate the host to generate neoantigens and trigger immune activation [65, 81]. Llosa NJ et al. indicated that the levels of PD-1, PD-L1, CTLA-4, LAG3, and IDO1 have been found to be significantly upregulated to balance the activated immune response in MSI-H tumors compared with MSS tumors [86]. Therefore, blocking the interaction between PD-1/PD-L1 and other immune negative regulatory pathways may increase activation of Th1 cytotoxic immune responses and significantly enhance the ability of the host to kill cancer cells, especially in dMMR/MSI-H tumors [87].

**Table 4:** The relationship between PD-L1 and dMMR/pMMR

Author/year	Tumor	*N*	PD-L1+ (%)	dMMR (%)	PD-L1^+^ in dMMR tumors (%)	PD-L1^+^ in pMMR tumors (%)	*P*	The impact of PD-L1 or dMMR on survival
*Gatalica Z* [74] 2014	CC	87	20.7	31.0	38.0	13.0	0.02	NA
*Inaguma S* [83] 2016	CRC	506	NA	NA	44.7	6.8	< 0.01	NA
*Inaguma S* [83] 2016	GC	180	NA	NA	46.7	12.1	< 0.01	NA
*Kim ST* [82] 2017	Advanced GI, GU, and others	430	16.5 in all, 28.6 in melanoma, 22.4 in GC, 20.9 in CRC, 12.5 in BTC, 7.1 in GU, 6.7 in HCC, 0.0 in pancreatic cancer and sarcoma	4.5 in all 7.1 in GC 6.7 in HCC 4.4 in CRC	38.9	15.2	< 0.01	*P* = 0.535 in GC *P* = 0.231 in mCRC *P* = 0.508 in sarcoma
*Mills AM* [26] 2018	Breast carcinoma	245	12.0 in all 32.0 in TNDC	0.04	100.0	NA	NA	NA
*Wang L* [84] 2018	GC	550	37.3	8.2	60.0	35.2	< 0.01	NA

*Abbreviations*: *N* number, *PD-L1* programmed death-ligand 1, *dMMR* mismatch repair deficient, *pMMR* mismatch repair proficient, *NA* not available, *CC* colon cancer, *CRC* colorectal cancer, *GC* gastric cancer, *GI* gastrointestinal cancer, *GU* genitourinary cancer, *BTC* biliary tract cancer, *HCC* hepatocellular carcinoma, *mCRC* metastatic colorectal cancer, *TNDC* triple negative ductal carcinoma

TMB is another promising predictor for anti-PD-1/PD-L1 immunotherapy compared to dMMR/MSI-H, and the relevant content has been widely studied in lung cancer [88–92], melanoma [93], bladder cancer [94], and others. The exploratory subgroup analyses in the CheckMate 026 trial demonstrated that patients with previously untreated stage IV or recurrent NSCLC obtained significant ORR and mPFS benefits from nivolumab than platinum-based chemotherapy in the high TMB (TMB ≥ 243 mutations) group (ORR 47% vs 28%; mPFS 9.7 months vs 5.8 months). No significant clinical benefit was observed in regards to ORR or PFS in the low (0 < TMB < 100 mutations) or medium TMB (100 ≤ TMB < 243 mutations) group [88]. In the CheckMate 568 trial, the ORR was 4%, 10%, 44%, and 39% when the TMB cutoffs were < 5, < 10, ≥ 10, and ≥ 15 mut/Mb in NSCLC patients treated with nivolumab plus ipilimumab as a first-line therapy. Subsequently, TMB ≥ 10 mut/Mb was regarded as the criteria to differentiate the high TMB and low TMB cohorts in the CheckMate 227 trial [89]. The prospective phase III trial confirmed that nivolumab plus ipilimumab resulted in a significantly longer PFS and higher ORR only in high TMB patients with stage IV or recurrent NSCLC compared with chemotherapy (mPFS 7.2 months vs 5.5 months; ORR 45.3% vs 26.9%) [90]. According to the abovementioned trials, the National Comprehensive Cancer Network guidelines firstly recommended that TMB was an emerging biomarker to identify patients with NSCLC for nivolumab or nivolumab plus ipilimumab in version 1 of 2019 [80]. The CheckMate 032 trial demonstrated better clinical benefit in high TMB (TMB ≥ 248 mutations) patients with SCLC [91]. Robert M. Samstein et al. reported that higher TMB was significantly associated with better OS in 1662 patients treated with either anti-CTLA-4 or anti-PD-1 therapies in diverse cancer types. These studies provided robust evidence for the predictive power of TMB in guiding the application of ICB [95]. TMB is commonly detected through tissue, and blood detection is the substitute due to the lack of tissue. Gandara et al. showed that high blood-based TMB (bTMB ≥ 16 mut/Mb) levels were positively associated with improved PFS and OS in NSCLC patients treated with atezolizumab versus docetaxel as a second-line or more-line choice [92]. More recently, Zhijie Wang et al. found that bTMB can be well estimated and measured by a cancer gene panel (CGP) named NCC-GP150 in patients with NSCLC. In clinical validation, 50 patients with NSCLC with high bTMB (≥ 6 mut/Mb) was associated with prolonged mPFS and higher ORR than patients with low bTMB (< 6 mut/Mb) (mPFS not reach vs 2.9 m; ORR 39.3% vs 9.1%) when treated with anti-PD-1/PD-L1 therapies [96] (see Table 5).

**Table 5:** TMB predicts the efficacy of ICB therapy

Clinical trial	Phase	Drug	TMB (mut/Mb)	Tumor	*N*	Response	PFS	OS
CheckMate026 [88]	III	Nivolumab	H ≥ 243	NSCLC	47	ORR 47%	*mPFS, 9.7 moths	1-year OS, 64%; mOS, 18.3 months
		Platinum-based CT			60	ORR 28%	*mPFS, 5.8 months	1-year OS, 60%; mOS, 18.8 months
		Nivolumab	L 0 to 99 and M 100 to 242		111	ORR 23%	mPFS, 4.1 months	1-year OS, 54%; mOS, 12.7 months
		Platinum-based CT			94	ORR 33%	mPFS, 6.9 months	1-year OS, 53%; mOS, 13.2 months
CheckMate568 [89]	II	Nivolumab + ipilimumab	< 5, 5–10, 10–15, ≥ 15	NSCLC	288	ORR 4%, ORR 10%, ORR 44%, ORR 39%	NA	NA
CheckMate227 [90]	III	Nivolumab + ipilimumab	≥ 10	NSCLC	139	ORR 45.3% 1-year DoR 68%	*1-year PFS, 42.6% *mPFS, 7.2 months	NA
		Platinum doublet CT			160	ORR 26.9% 1-year DoR 25%	*1-year PFS, 13.2% *mPFS, 5.5 months	
		Nivolumab + ipilimumab	< 10				mPFS, 3.2 months	
		Platinum doublet CT					mPFS, 5.5 months	
CheckMate 032 [91]	Exploratory	Nivolumab + ipilimumab	H ≥ 248	SCLC	26	ORR 46.2%	1-year PFS, 30.3% mPFS, 7.8 months	1-year OS, 62.4%; mOS, 22.0 months
		Nivolumab			47	ORR 21.3%	1-year PFS, 21.2% mPFS, 1.4 months	1-year OS, 35.2%; mOS, 5.4 months
		Nivolumab + ipilimumab	M 143 to 247		25	ORR 16.0%	1-year PFS, 8.0% mPFS, 1.3 months	1-year OS, 19.6%; mOS, 3.6 months
		Nivolumab			44	ORR 6.8%	1-year PFS, 3.1% mPFS, 1.3 months	1-year OS, 26.0%; mOS, 3.9 months
		Nivolumab + ipilimumab	L 0 to 143		27	ORR 22.2%	1-year PFS, 6.2%; mPFS, 1.5 months	1-year OS, 23.4%; mOS, 3.4 months
		Nivolumab			42	ORR 4.8%	1-year PFS, NC;mPFS, 1.3 months	1-year OS, 2.1%; mOS, 3.1 months
POPlAR [92]	Training	Atezolizumab	H-bTMB ≥ 16	NSCLC	25	NA	*mPFS, 4.2 months	*mOS, 13.0 months
		Docetaxel			38		*mPFS, 2.9 months	*mOS, 7.4 months
OAK [92]	Validation	Atezolizumab	H-bTMB ≥ 16	NSCLC	77	ORR 21%	*PFS (HR 0.65, 95% CI 0.47–0.92; *P* = 0.013)	*mOS, 13.5 months
		Docetaxel			81	ORR 10%		*mOS, 6.8 months
		Atezolizumab	bTMB < 16	NSCLC	216	ORR 13%	PFS (HR 0.98, 95% CI 0.80–1.2)	OS (HR 0.65, 95% CI 0.52–0.81)
		Docetaxel			209	ORR 12%		
Zhijie W et al. [96]		Anti-PD-1/PD-L1 therapies	bTMB ≥ 6 bTMB < 6	NSCLC	28 22	*ORR 39.3% *ORR 9.1%	*mPFS: NR *mPFS, 2.9 months	

*Abbreviations: N* number, *ICBs* immune checkpoint blockades, *PD-L1* programmed death ligand 1, *PD-1* programmed death-1, *CTLA-4* cytotoxic T lymphocyte antigen-4, *mAb* monoclonal antibody, *NSCLC* non-small cell lung cancer, *SCLC* small-cell lung cancer, *CT* chemotherapy, *ORR* objective response rate, *DoR* duration of response, *PFS* progression-free survival, *mPFS* median progression-free survival, *OS* overall survival, *mOS* median overall survival, *TMB* tumor mutation burden, *H* high, *M* medium, *L* low, *bTMB* blood-based tumor mutation burden, *mut* mutation, *mut/Mb* mutation per megabase, *Ref* reference, *vs* versus, *NR* not reached, *NA* not available

*Denotes the difference was statistically significant

Compared with dMMR/MSI-H or PD-1/PD-L1 expression, the TMB is emerging as a more accurate, comprehensive, and compelling potential biomarker that could predict the efficacy of ICB therapy. However, there is no consensus on the measuring of TMB status [95]. In several studies, most patients with MSI-H had high TMB levels; however, not all patients with high TMB levels had dMMR/MSI-H status or high PD-L1 expression [88, 97, 98]. Rizvi H et al. indicated that there was no correlation between PD-L1 and TMB status in patients with NSCLC treated with ICB (*r* = 0.1915, *P* = 0.08). Patients with high-TMB and positive PD-L1 expression had the highest rate of durable clinical benefit than that with only one or neither variable presence (50% vs. 18.2–35.5%) [98]. Fabrizio DA et al. demonstrated that 99.7% of CRC patients with MSI-H had high TMB status (6.3–746.9 mut/Mb); meanwhile, 97.0% of CRC patients with MSS were low TMB (0.0–10.8 mut/Mb) in a large population. Although there was a high consistency between MSI-H and high TMB status in CRC, 2.9% (163/5702) of patients with MSS were still considered as high TMB [97]. Zachary R et al. analyzed 100,000 human cancer genomes to reveal the landscape of TMB and found that nearly 83% of tumor samples with MSI-H showed high TMB status (TMB > 20 mut/Mb), whereas only 16% of tumor samples with high TMB was MSI-H and nearly 84% were classified as MSS [99]. ICB therapy is not recommended for patients with MSI-L/MSS, but these patients might have high TMB and could still benefit from ICB therapy [99]. The co-occurrence of high TMB and MSI-H varied among diverse cancer types, and they usually come together in gastrointestinal cancers, but are rarely consistent in lung cancer or melanoma in which the presence of high TMB is common [99]. Comprehensive analysis of dMMR/MSI-H, PD-L1, and TMB or a multivariable predictive model composed of 9 exome parameters (the DNA repair pathway status, the WNT pathway status, the number of TCR clones, the number of neoantigens, the HLA*A*1 and HLA*A*24 status, and the fractions of signatures 1A, 1B, and 6) resulted in greater predictive power and may allow for the optimal usage of ICB therapy [88, 98, 100, 101].

## Conclusions and prospects

Immunotherapy has dramatically changed the therapeutic landscape of multiple tumors and has boosted enthusiasm regarding cancer treatment. Recent positive results from clinical trials of ICB therapies alone or in combination for “difficult-to-treat” dMMR/MSI-H tumors have led to great hope for immunotherapy application in this specific population. dMMR/MSI-H has been approved by the FDA as an indication of ICB for metastatic cancers, irrespective of the cancer types, presumably due to the enhanced immune response through the presence of increased somatic mutations and “nonself” neoantigens in these tumors. The novel use of ICB therapies as first-line or neoadjuvant treatments in dMMR/MSI-H tumors may have the potential to expand the indications. dMMR/MSI-H has its unique advantages compared with PD-L1, TMB, TILs, and other new predictors. Despite encouraging results of ICB by recognizing dMMR/MSI-H, only a fraction of patients typically have the dMMR/MSI-H features, and some sensitive patients still cannot be distinguished. Comprehensive analysis of multiple markers will provide the optimal strategy to identify sensitive patients to ICB therapy in the near future.

## Additional files

Additional file 1: **Figure S1.** Statistically significant survival analysis of different tumors between the dMMR group and the pMMR group. (PPTX 379 kb)

Additional file 2: **Figure S2.** Survival analysis of tumors with or without MMR gene alterations. (PPTX 2269 kb)

## Data Availability

Not applicable.
