# Supplementary material for: Mismatch repair deficiency/microsatellite instability-high as a predictor for anti-PD-1/PD-L1 immunotherapy efficacy
Source: J Hematol Oncol. 2019 May 31;12:54. doi: 10.1186/s13045-019-0738-1 (PMC6544911; doi:10.1186/s13045-019-0738-1)
Supplement: Supplementary file 1 — Figure S1. Statistically significant survival analysis of different tumors between the dMMR group and the pMMR group. (PPTX 379 kb) [file 13045_2019_738_MOESM1_ESM.pptx]

## Slide 1
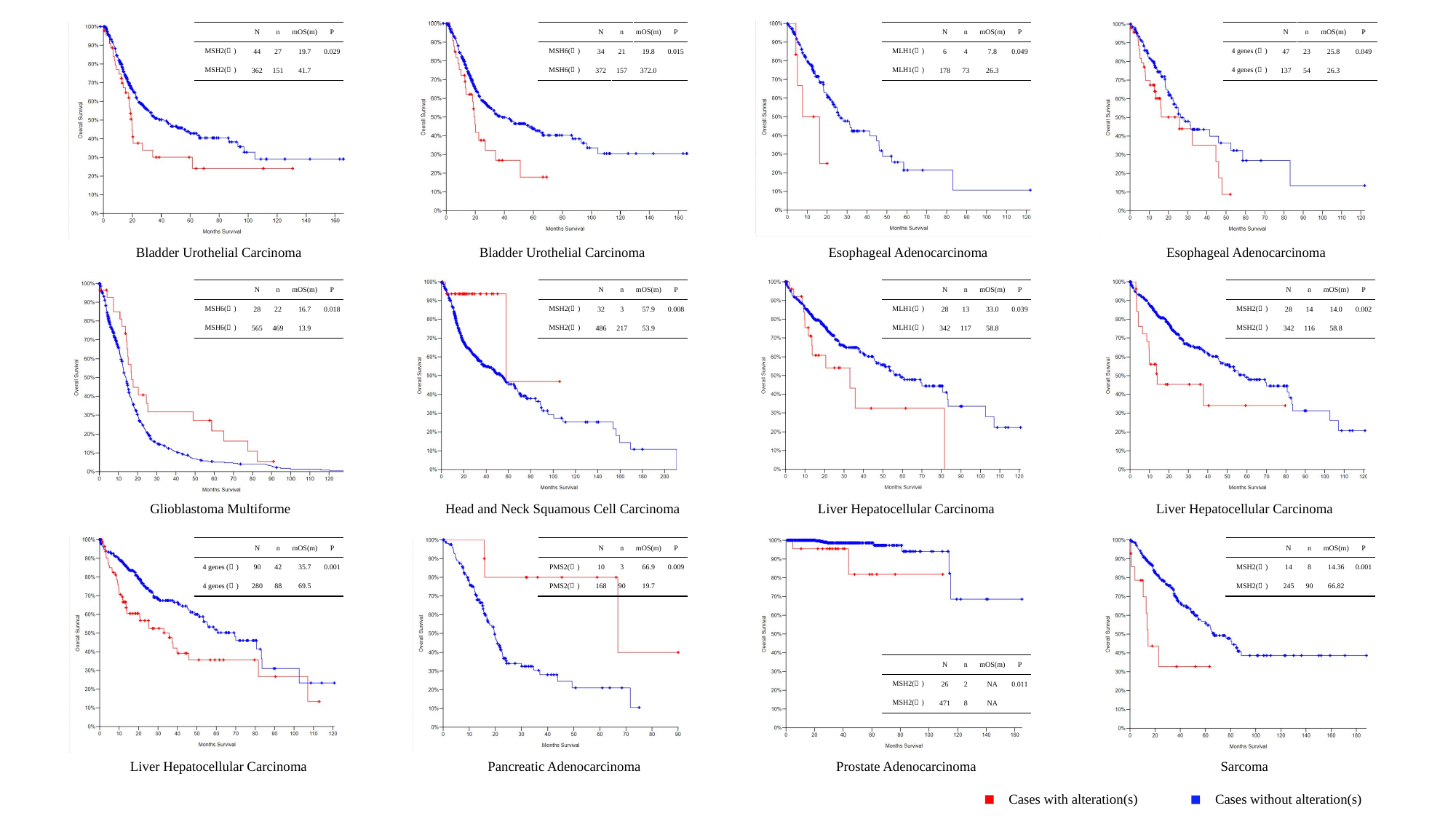

Bladder Urothelial Carcinoma
Bladder Urothelial Carcinoma
Esophageal Adenocarcinoma
Esophageal Adenocarcinoma
Glioblastoma Multiforme
Head and Neck Squamous Cell Carcinoma
Liver Hepatocellular Carcinoma
Liver Hepatocellular Carcinoma
Liver Hepatocellular Carcinoma
Pancreatic Adenocarcinoma
Prostate Adenocarcinoma
Sarcoma
Cases with alteration(s)
Cases without alteration(s)
