# Supplementary material for: Mismatch repair deficiency/microsatellite instability-high as a predictor for anti-PD-1/PD-L1 immunotherapy efficacy
Source: J Hematol Oncol. 2019 May 31;12:54. doi: 10.1186/s13045-019-0738-1 (PMC6544911; doi:10.1186/s13045-019-0738-1)
Supplement: Supplementary file 2 — Figure S2. Survival analysis of tumors with or without MMR gene alterations. (PPTX 2269 kb) [file 13045_2019_738_MOESM2_ESM.pptx]

## Slide 1
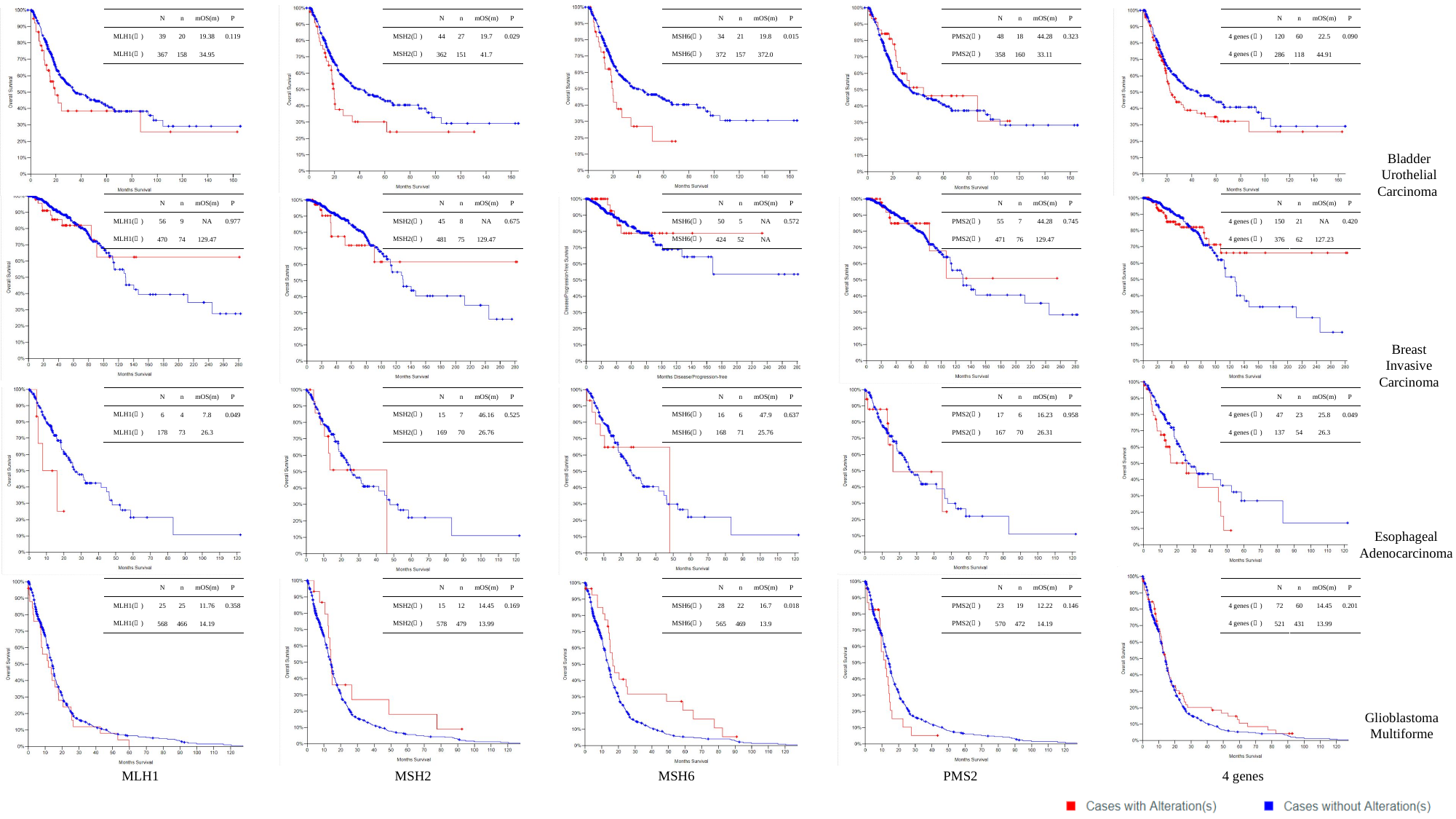

Bladder Urothelial Carcinoma
Breast Invasive Carcinoma
Esophageal Adenocarcinoma
MSH6
Glioblastoma Multiforme
MLH1
MSH2
PMS2
4 genes

## Slide 2
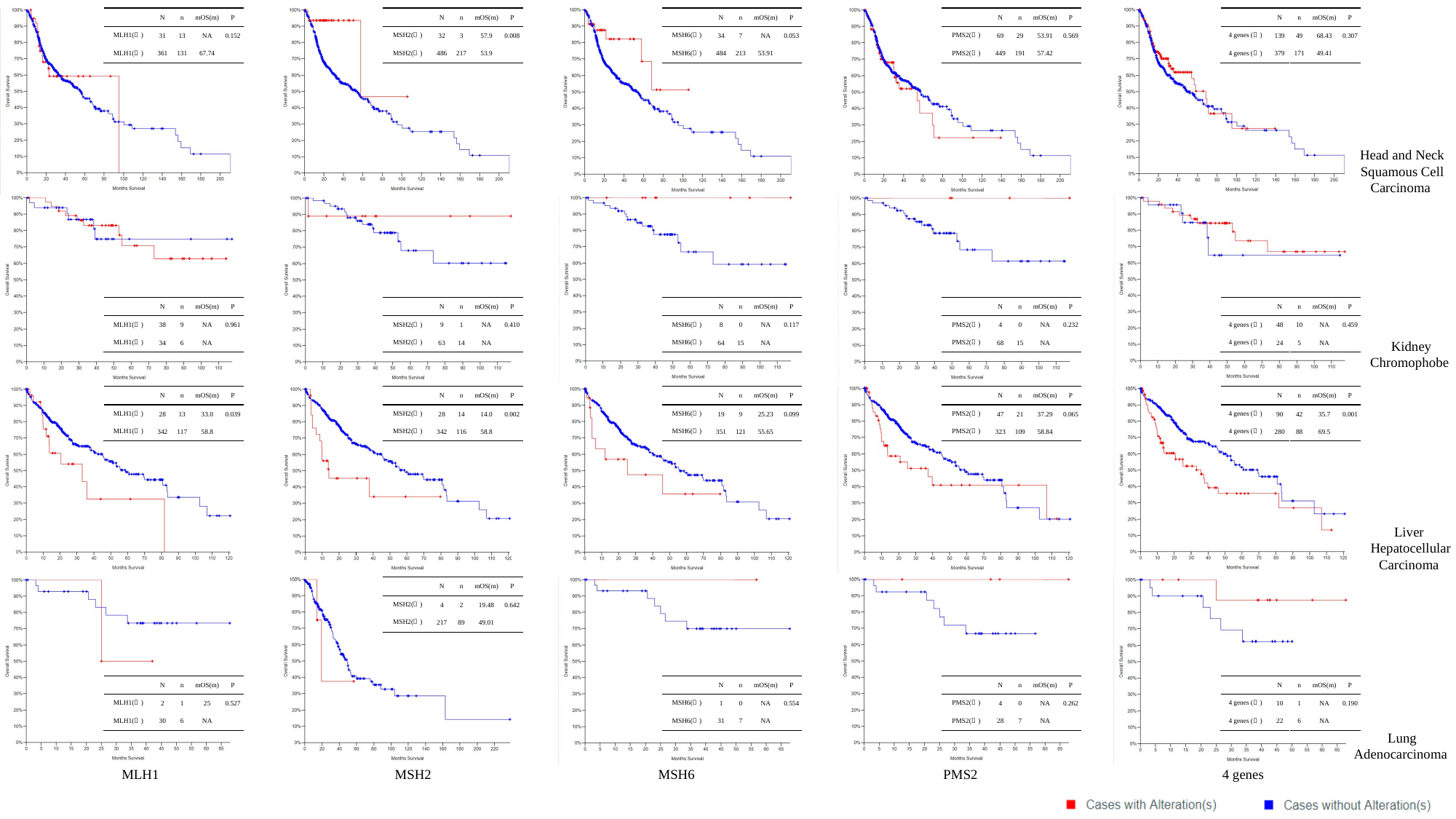

Head and Neck Squamous Cell Carcinoma
Kidney Chromophobe
Liver
Hepatocellular Carcinoma
Lung Adenocarcinoma
MLH1
MSH2
MSH6
PMS2
4 genes

## Slide 3
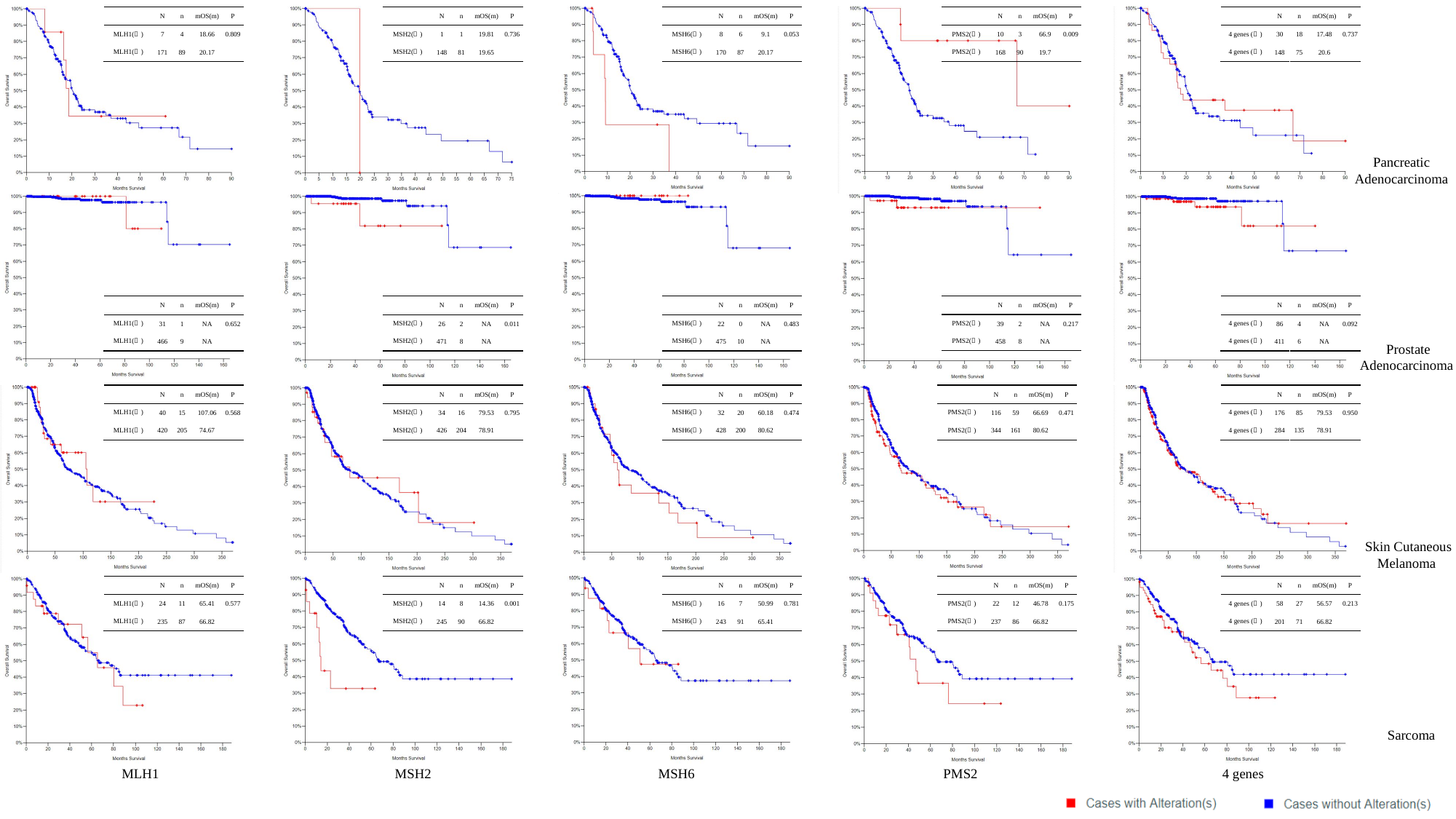

Pancreatic Adenocarcinoma
Prostate Adenocarcinoma
Skin Cutaneous Melanoma
MSH2
Sarcoma
MLH1
MSH6
PMS2
4 genes

## Slide 4
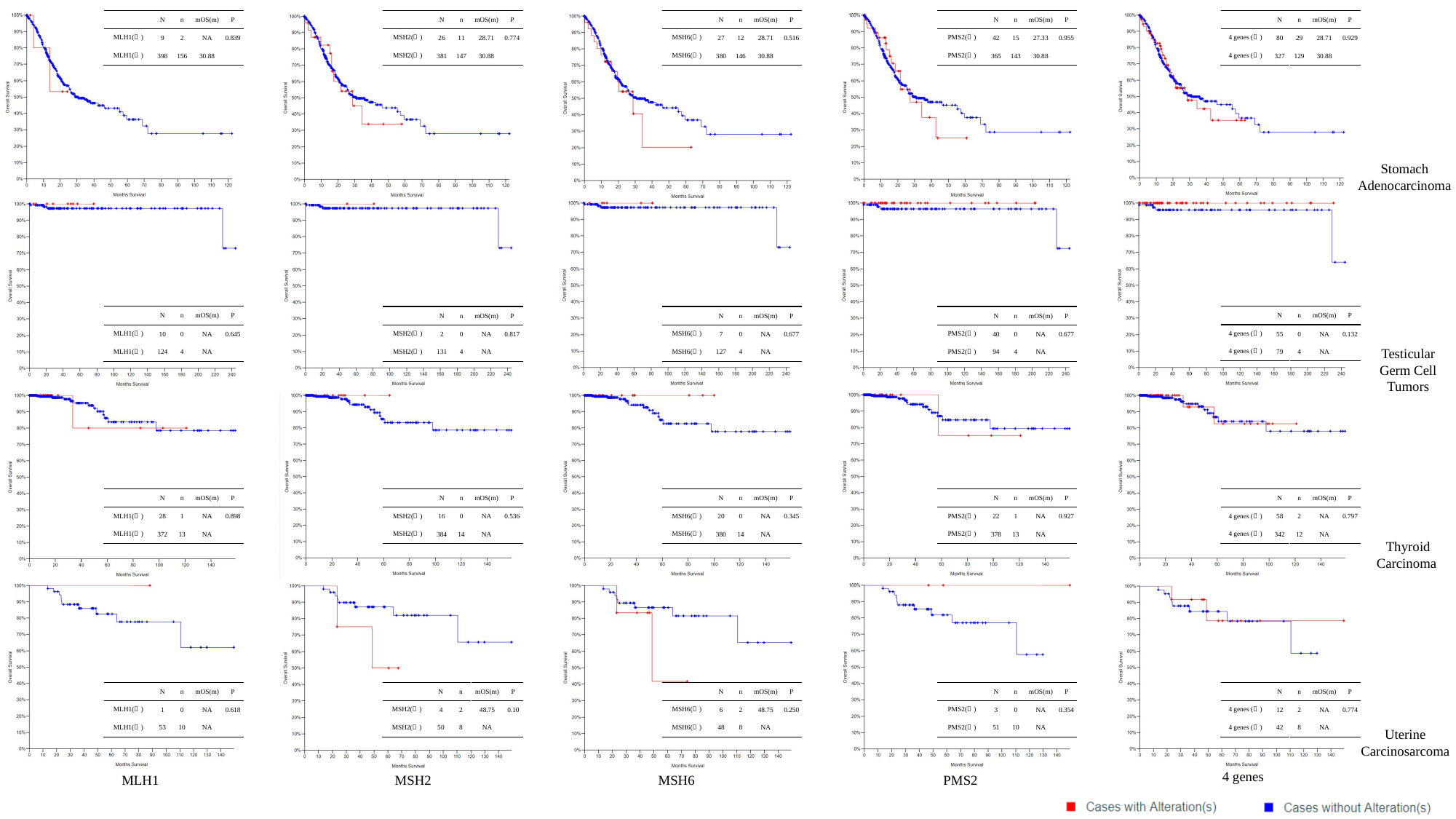

MLH1
MSH2
MSH6
PMS2
Stomach Adenocarcinoma
Testicular Germ Cell Tumors
Thyroid Carcinoma
Uterine Carcinosarcoma
4 genes
